# Surgeons for the Public Health and Marine Hospital Service

**Published:** 1912-03

**Authors:** 


### Abstracts and Selections.

### Surgeons for the Public Health and Marine Hospital Service.

TREASURY DEPARTMENT,
BUREAU OF PUBLIC HEALTH AND MARINE HOSPITAL SERVICE.

WASHINGTON, January 30, 1912.

A board of commissioned medical officers will be convened to meet at the Bureau of Public Health and Marine Hospital Service, 3 B Street, S. E., Washington, D. C., Monday, April 8, 1912, at 10 o'clock a. m., for the purpose of examining candidates for admission to the grade of assistant surgeon in the Public Health and Marine Hospital Service.

Candidates must be between 22 and 30 years of age, graduates of a reputable medical college, and must furnish testimonials from responsible persons as to their professional and moral character.

The following is the usual order of the examinations: 1. Physical; 2, oral; 3, written; 4, clinical.

In addition to the physical examination, candidates are required to certify that they believe themselves free from any ailment which would disqualify them for service in any climate.

The examinations are chiefly in writing, and begin with a short autobiography of the candidate. The remainder of the written exercise consists in examination of the various branches of medicine, surgery, and hygiene.

The oral examination includes subjects of preliminary education, history, literature, and natural sciences.

The clinical examination is conducted at a hospital, and, when practicable, candidates are required to perform surgical operations on a cadaver.

Successful candidates will be numbered according to their attainments on examination, and will be commissioned in the same order as vacancies occur.

Upon appointment the young officers are, as a rule, first assigned to duty at one of the large hospitals, as at Boston, New York, New Orleans, Chicago, or San Francisco.

After four years' service, assistant surgeons are entitled to examination for promotion to the grade of passed assistant surgeon.

Promotion to the grade of surgeon is made according to seniority and after due examination, as vacancies occur in that grade.

Assistant surgeons receive $1600, passed assistant surgeons $2000, and surgeons $2500 a year. When quarters are not provided, commutation at the rate of $30, $40, and $50 a month, according to grade, is allowed.

All grades above that of assistant surgeon receive longevity pay, 10 per cent in addition to the regular salary for every five years' service up to 40 per cent after twenty years' service.

The tenure of office is permanent. Officers traveling under orders are allowed actual expenses.

For further information, or for invitation to appear before the board of examiners, address Surgeon General, Public Health and Marine Hospital Service, Washington, D. C.

---

The transperitoneal route, with retraction of the colon, affords the best exposure in radical nephrectomy for neoplasm.—*American Journal of Surgery.*

---

Indiana State Board of Examiners Sues Healers.—Recently the Indiana State Board of Examiners filed suits against a number of "chiropractors," a sect of "healers" who practice after a course of study of only a few months, on the ground that they are practicing the healing art without complying with the law. On the other hand, the chiropractors claim that they should be exempt from the provision of the law because their name is not contained in the statute.—*Exchange.*

In injuries to the cord, if the tendon reflexes are preserved, even slightly, the surgeon may exclude complete and irremediable severance of the cord; but the total loss of these reflexes during the first few days is not conclusive, as the loss may be transitory. —*American Journal of Surgery.*

